# Securing Mobile Ad Hoc Networks Using Danger Theory-Based Artificial Immune Algorithm

**DOI:** 10.1371/journal.pone.0120715

**Published:** 2015-05-06

**Authors:** Maha Abdelhaq, Raed Alsaqour, Shawkat Abdelhaq

**Affiliations:** 1 School of Computer Science, Faculty of Information Science and Technology, University Kebangsaan Malaysia (UKM), 43600, Bangi, Selangor, Malaysia; 2 Business Administration Faculty, Indiana Wesleyan University, 4201 S. Washington St, Marion, Indiana 46953, United States of America; Semmelweis University, HUngary

## Abstract

A mobile ad hoc network (MANET) is a set of mobile, decentralized, and self-organizing nodes that are used in special cases, such as in the military. MANET properties render the environment of this network vulnerable to different types of attacks, including black hole, wormhole and flooding-based attacks. Flooding-based attacks are one of the most dangerous attacks that aim to consume all network resources and thus paralyze the functionality of the whole network. Therefore, the objective of this paper is to investigate the capability of a danger theory-based artificial immune algorithm called the mobile dendritic cell algorithm (MDCA) to detect flooding-based attacks in MANETs. The MDCA applies the dendritic cell algorithm (DCA) to secure the MANET with additional improvements. The MDCA is tested and validated using Qualnet v7.1 simulation tool. This work also introduces a new simulation module for a flooding attack called the resource consumption attack (RCA) using Qualnet v7.1. The results highlight the high efficiency of the MDCA in detecting RCAs in MANETs.

## Introduction

In the last few decades, many researchers have focused on mobile ad hoc networks (MANETs) as wireless networks with specific features not found in other network types. The decentralization, rapid deployable topology, and open wireless medium of MANETs increase their feasibility for application in roughly structured areas, such as earthquake-prone areas and war-torn regions. However, these features as well as the limitations of MANET (i.e., sharing of channel bandwidth and the limitation in the energy of nodes) make this network very vulnerable to different types of attacks. Many intrusion detection systems (IDSs) have been introduced to protect the routing protocols in MANETs [1–3]. However, the conventional cryptographic IDSs utilized to secure routing protocols in MANETs increase the control overhead by transmitting extra security information (digital signatures and function hashes) through routing packets. A valid digital signature gives a destination reason to believe that the packet was created by a known sender. For hashing function, most of the work done around using hashing techniques in packets authentication and route table entries.

Moreover, the lack of a fixed infrastructure in MANETs renders the use of certificate authorities infeasible. Thus, the general trend at present is to employ lightweight computing algorithms to secure MANETs [4].

On the basis of the many similarities between the human body tissue environment and the MANET environment concluded from the study in [5], we claim that the robust defense achieved by the human immune system (HIS) can be translated into an artificial immune system (AIS) to protect MANETs. AISs are defined as a set of computational algorithms or theories that reflect one or more HIS concepts and principles [6]. The introduced AIS intrusion detection algorithms can detect attacks in a decentralized and self-organizing manner, which means that central management points in the security system are not necessary when AISs are applied. This advantage renders the technique feasible for securing MANETs and addressing the limitations and challenges of such networks.

Aickelin et al. [7] attempted to improve the performance of previously introduced AISs and established the “danger project” [7,8], which is primarily based on danger theory in immunology. Danger theory implies that the response of the immune system to incoming pathogens is based mainly on the existence of danger or safe signals emitted from the body tissues and caused by these pathogens [9,10]. In a danger project, a group of computer scientists and immunologists map actual up-to-date immunology into the AIS [11–13]. The dendritic cell algorithm (DCA) is one of the most well-known contributions to the danger project. This algorithm utilizes the role of dendritic cells (DCs) in the HIS as forensic navigators and important anomaly detectors. DCs are defined as antigen-presenting cells in innate immunity; these cells either stimulate or suppress T-cells in adaptive immunity, thereby controlling the type of response of the immune system [6].

The objective of this paper is to propose and investigate the capability of a danger theory-based artificial immune algorithm called the mobile dendritic cell algorithm (MDCA) to detect flooding-based attacks in MANETs. The MDCA applies the dendritic cell algorithm (DCA)[5] to secure the MANET with additional improvements. The MDCA is tested and validated using Qualnet v7.1 simulation tool. This work also introduces a new simulation module for a flooding attack called the resource consumption attack (RCA) using Qualnet v7.1.

This paper is organized as follows. Section 2 reviews the background of the study. Section 3 thoroughly explains the MDCA. Section 4 describes the simulation design and environment applied and the details of the proposed MDCA validation and testing. Finally, Section 5 discusses conclusions and future works.

## Research Background

### Dendritic Cells

DCs have three main differentiation states, immature, semi-mature and mature. When immature DCs receive enough input signals, they become either semi-mature or mature DCs based on the concentration of specific types of these input signals. Immature DCs receive four types of input signals, PAMP, danger, safe and inflammation signals. PAMP signals indicate strongly the existence of infectious pathogen. Danger signals are released by necroses which are the human body cells under stress or abnormal death. However, safe signals are released by apoptosis which are healthy cells or cells that die in a normal way. Inflammation signals are released as a result of an increase in the cells’ temperature caused from unhealthy state or infection. DCs input signals are divided into, endogenous and exogenous signals. Endogenous signals are those released from the cells of the body itself such as safe, danger and inflammation signals. However, exogenous signals are the signals released from the microbes which inter the human body from the outside environment. An example of this type is PAMP signals [14].

When immature DCs are exposed to these input signals, the concentration of each controls their next terminal differentiation state (either mature or semi-mature DCs). For example, if the concentration of the received PAMP signals and danger signals are greater than that of safe signals, this means the differentiation of immature DCs is to mature DCs. PAMP and danger signals cause the receiver immature DCs to process its contents and produce a certain cytokine called interleukin-12 (IL-12). Also, PAMP and danger signals induce immature DCs to produce costimulatory molecules (csm), also called CD80/86 in biology. The csm signal simplifies the process of antigen presentation to the T-cells in lymph nodes. Conversely, if the concentration of safe signals is greater than that of PAMP and danger signals, then immature DCs should differentiate to semi-mature DCs. Also, safe signals are responsible for producing interleukin-10 (IL-10) in this case. Additionally, safe signals induce producing csm signals by the DCs same as PAMP and danger signals. Therefore, the received input signals indicate the behavioral context of the digested antigens if either they are benign or malignant.

**Fig 1:**
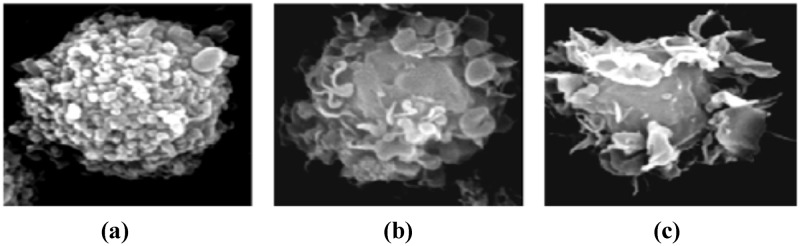
States of DC differentiations. Immature (A), semi-mature (B), mature (C) [12].

Fig 1 pictures the three differentiation states of DCs. Although DCs have same receptor structure in the three differentiation states; they appear different in their morphology. As noticed in Fig 1(b) and 1(c), semi-mature and mature DCs have wider surfaces than immature DC. The reason behind that refers to increasing the capability of both mature and semi-mature DCs to show their receptors and bind with T-cells’ receptors when they are encountered in lymph nodes.

### Flooding Attack on AODV

**Fig 2:**
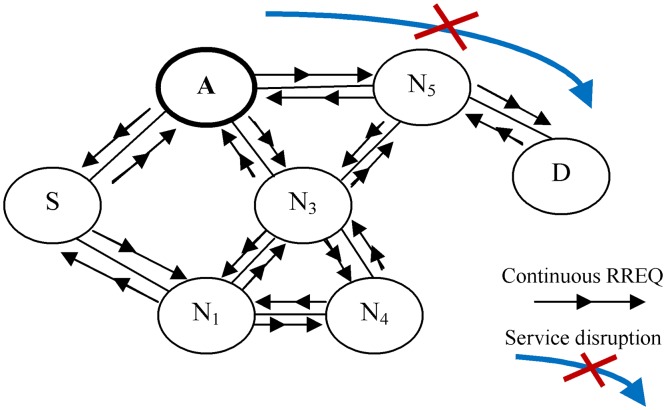
Propagation of RREQ packet in AODV. S: source node, D: destination node, N1 to N5: intermediate nodes.

The AODV, the underlying routing protocol considered in this study, is a reactive on-demand routing protocol in MANETs. The AODV has many advantages, such as self-starting ability, the capability to avoid congested routes, and the feasibility over scalable wireless networks [15]. However, the dependency of the AODV on broadcasting route request (RREQ) packets in the discovery process renders its protocol susceptible to RCAs [16]. Fig 2 shows the propagation of the RREQ packet in the AODV in a normal case (no attacks). When a source node S needs to discover a new route to the destination node D, it simply broadcasts the RREQ packet to all of its neighboring nodes and requests these nodes to reply with a fresh enough route to node D if such route is available in their routing tables. Otherwise, the neighboring nodes have to rebroadcast the received RREQ packet to their neighboring nodes. The process continues until either an intermediate node or the destination node D itself replies with a fresh enough route by unicasting a route reply (RREP) packet to source node S.

**Fig 3:**
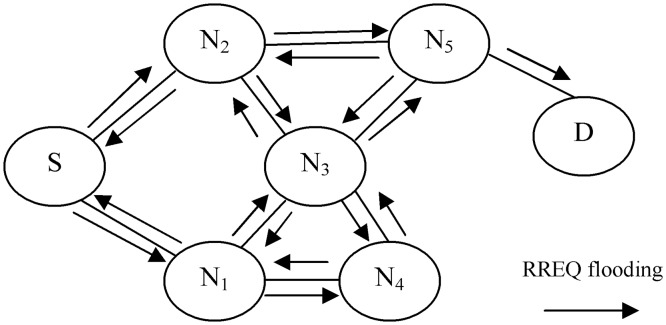
RREQ continuously broadcasted by RCA. S: source node, D: destination node, N1 to N5: intermediate nodes.

Based on the AODV routing protocol, source node S must wait for the timeout period to receive RREP from either an intermediate node or the destination node D. Otherwise, source node S can rebroadcast the RREQ packet with high timeout to receive the desired RREP packet with a newly established route to the destination node D. In addition, based on the AODV routing protocol, the RREQ packet must contain two important fields; namely, the RREQ packet broadcast identification (ID) and the source node IP address. The two fields distinguish each RREQ packet from other packets broadcasted by the same source node to discover different routes. When intermediate nodes receive duplicate RREQ packets (packets with the same IP address and broadcast ID), they simply discard such packets. In this way, the AODV helps the legitimate nodes avoid network overflow and unnecessary power consumption. However, in RCA (Fig 3), the attacker node A utilizes the broadcasting stage in the AODV and keeps flooding the network with fake RREQ packets with different broadcast IDs and source node IP addresses. The main purpose of RCA is to consume the energy of legitimate nodes and the available link throughput. If RCA has been carried out through a cooperation between groups of attackers, a period spanning a few minutes is enough to stop the normal connections of a network and consume its resources.

### Existing Artificial Immune ID Systems

The work in [17,18] introduced the first studies that utilized AIS for MANETs [19]. The detection is applied over a network layer. The authors relied on a co-stimulation concept represented by a danger signal to gather information about the packet loss in the connection path.

In [20], the author proposed a Network threat recognition with immune inspired anomaly detection (NetTRIIAD) model. The NetTRIIAD model utilizes danger theory and implements negative selection in a different way than that used in previous self/non-self-dependent studies. The NetTRIIAD model is used to detect denial of service (DoS) attack, dropping packets, and delaying packets over wired networks. It is a prototype that does not have enough robustness to protect real wired networks. In addition, this model cannot work properly when applied in the scaling problem. It also lacks a good self-set used in the negative selection operation.

Greensmith et al. [11,12] introduced a DCA based on empirical studies on DCs in immunology. However, the dependency of the algorithm on the order of the examined antigens makes it vulnerable when a mix of benign and malignant antigens has been examined. Therefore, DCA results have high false positive (FP) and false negative (FN) rates. Wallenta et al. [21] used the DCA to detect cache poisoning attacks over a wireless sensor network. Drozda et al. [22] proposed a co-stimulation inspired approach (CIA) for MANETs. The CIA detects three types of attacks, namely, wormhole attack, dropping packet attack, and packet delay. Despite its efficiency, CIA has many drawbacks, the most crucial of which is its dependency on a watchdog. This drawback has caused failures in many cases [23]. Ou [13] combined the features of the DCA and the agent-based IDS to introduce an agent-based AIS (ABAIS), the aim of which is to detect viruses and worms over the Internet.

## Mobile Dendritic Cell Algorithm Design

### MDCA Specifications

The MDCA is a standalone algorithm designed to secure routing protocols in each mobile node in MANETs. This algorithm depends mainly on the function of DCs as anomaly detectors and immune response controllers. Therefore, each DC in the MDCA is responsible for two main tasks: detecting RCA in the AODV and controlling the response of each mobile node to the detected RCA. Specifically, each DC passes through three main stages: (i) collection stage, (ii) processing stage, and (iii) response stage. Unlike the DCA, the MDCA in the collection stage does not use antigens and signals matrix; instead each immature DC (iDC) collects only one antigen and its related signals.

**Fig 4:**
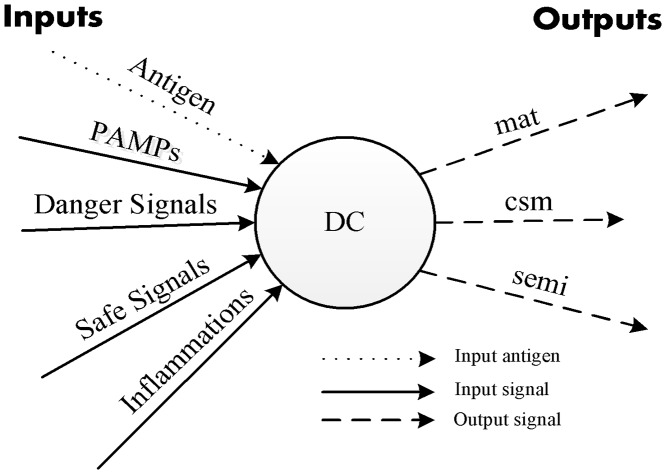
Main inputs and outputs of DC.

As shown in Fig 4, the main inputs of each DC in the MDCA are the input antigen and a set of its input signals, including PAMP, danger, safe, and inflammation signals. However, similar to those in the DCA, the main outputs of each DC in the MDCA include costimulatory molecules (*csm*); interleukin-10 (IL-10), which causes the iDC to transform into a semi-mature DC (smDC); and interleukin-12 (IL-12), which causes the iDC to transform into a fully mature DC (mDC) [12]. IL-10 and IL-12 are called *semi* and *mat*, respectively.

Input signals differ from one another in terms of strength and the indication of an existence of an attack. For example, a PAMP signal strongly indicates the existence of an attack and induces the production of *mat* and *csm* output signals. A danger signal also indicates the existence of an attack, but its strength is lower than that of the PAMP signal. A danger signal induces the production of *mat* and *csm* signals as well. A safe signal strongly indicates the non-existence of an attack and induces the production of *semi* and *csm* signals. Inflammation signals are considered as an amplifier for the other input signals. They are not applied in the detection of RCAs in this work; since there are no signals which can be applied to represent this type of signal.

Three input signals are applied in this work to detect RCAs in MANETs: PAMP, danger, and safe signals. As PAMP and safe signals are two strong signals that provide opposite indications and effects, the MDCA applies both of them to represent the rate at which the RREQ is received by the destination node in the MANET. In the AODV routing protocol [24], the rate at which the RREQ packet is received from the same source node cannot exceed 3 times/s in the normal case (zero attackers). Accordingly, the MDCA calculates the concentration of the PAMP signal to represent the RREQ rate that is equal to or greater than 4 times/s and the safe signal lower than 4 times/s.

However, a danger signal represents an increase in the number of link breaks registered by the mobile node that applies the MDCA. As the number of link breaks in normal states (no congestion and no attack) does not exceed 3 times/s, the MDCA calculates the concentration of the danger signal to represent the number of link breaks per second which exceeds 3 times/s. The number of link breaks is chosen to represent the danger signal because such value does not strongly indicate the existence of an attack. In other words, link breaks may increase because of normal congestion in the network itself.

The MDCA processes the infusion of each antigen signal using the Eqs 1, 2, and 3 as well as the weights in Table 1 to calculate the concentration of three main output signals (*csm*, *mat*, and *semi*, respectively) for each DC:

$$csm=\frac{\left(2\times Cp)+(1\times Cs)+(2\times Cd\right)}{\left(2+1+2\right)}$$ (1)

$$mat=\frac{\left(2\times Cp)+(1\times Cs)+(-2\times Cd\right)}{\left(2+1-2\right)}$$ (2)

$$semi=\frac{\left(0\times Cp)+(0\times Cs)+(3\times Cd\right)}{\left(0+0+3\right)}$$ (3)

where Cp, Cd, and Cs are the calculated concentrations for the PAMP, danger, and safe signals, respectively.

**Table 1:** Weights for processing input signals.

	PAMP	Danger	Safe
***csm***	2	1	2
***semi***	0	0	3
***mat***	2	1	-2

In the MDCA, the weight value of the safe signal for calculating the *mat* signal is changed from (-3) as in [12] to (-2) for mathematical purposes. Each DC continues calculating the accumulative values for each output signal related to a certain antigen until the *csm* exceeds a certain threshold (15 in the experiments conducted in this work) and until iDC size is exceeded. The MDCA is distinguished from the DCA in terms of iDC size because the MDCA considers the size of the DC to be equal to 1 in all of its antigens and signal manipulations; here, iDC size refers to the number of antigens processed by the iDC in each time period.

**Fig 5:**
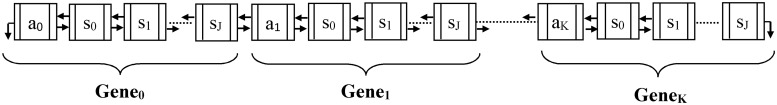
Genes list in the Genes Store.

**Fig 6:**
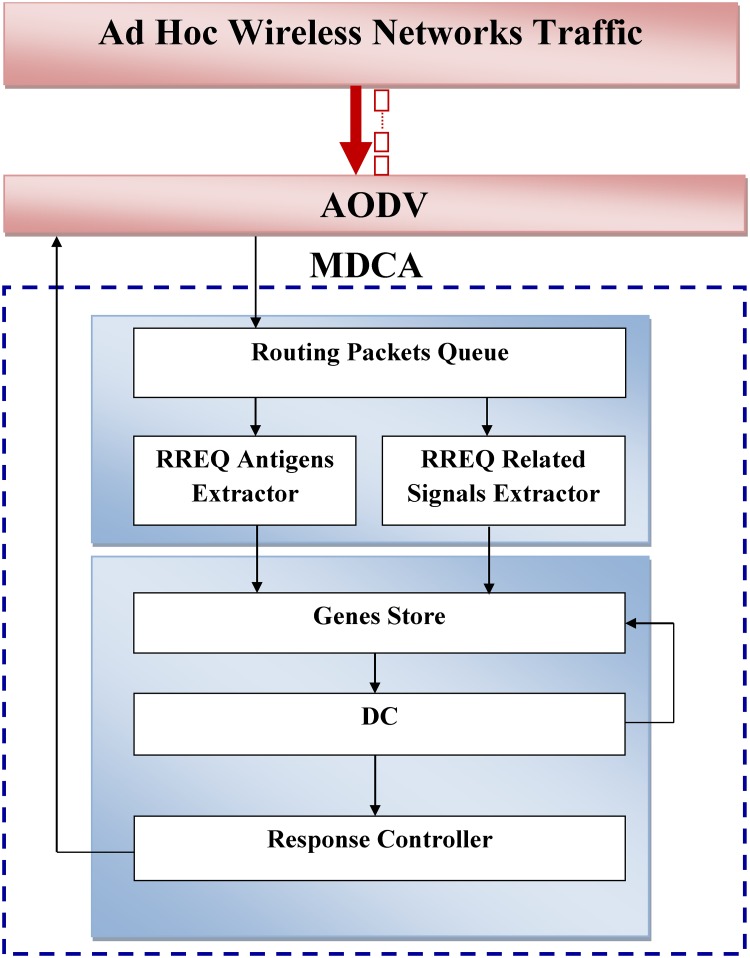
MDCA model.

Establishing the correlation between each input antigen and its related input signals is essential in the MDCA to avoid high FP and FN rates resulting from the DCA. The correlation is built using a genes list that represents the input antigens and their related input signals. The list depicted in Fig 5 represents the order of each input antigen and its related signals in the Genes Store of the MDCA model shown in Fig 6. The Genes Store stores K number of antigens, each of which may be correlated with J signals. Processing only one antigen with its related signals (gene) has many advantages; for example, processing a certain set of signals generates the context of their related antigen and does not wrongly affect the resulting context of other antigens.

### MDCA Model

Fig 6 explains the MDCA model and its interaction with the AODV routing protocol that receives the RREQ packets from the mobile nodes in MANET. The MDCA is designed to be plugged into the network layer of each mobile node. The MDCA comprises six main components: routing packet queue, RREQ antigen extractor, RREQ related signal extractor, Genes Store, DC, and response controller. When the AODV receives RREQ, it must first check the context of such RREQ to determine its origin, that is, whether the RREQ is sent by an attacker or a legitimate source node. Therefore, the MDCA secures the AODV by processing fake RREQs that consume the resources of the destination nodes and the network itself. The MDCA also performs local anomaly intrusion detection in each mobile node without making any information cooperation between the mobile nodes in the MANET.

The MDCA stores the received RREQs in the routing packet queue and processes them according to the first in, first out queuing system. Then, the MDCA extracts the RREQ antigen and its input signals (PAMP, safe, and danger) using the RREQ antigen extractor and the RREQ related signal extractor, respectively. In the RREQ antigen extractor, the extracted antigen represents the IP address of the source node. Meanwhile, the RREQ related signal extractor calculates the PAMP, safe, and danger signals based on the behavior of the processed RREQ in terms of its rate and the link breaks it caused.

The antigen of each RREQ and its related signals are combined in the genes list in the Genes Store. The existence of a new gene stimulates the MDCA to create a new DC in the DC component that checks the genes list continuously for new input genes. Each DC manipulates only one antigen but not only one gene. Specifically, the Genes Store may store genes with the same antigen but different signals. However, whether a certain input antigen can be duplicated in several genes in the genes list is not a condition in this process.

Once a DC is created, it manipulates the newly created gene in the genes list. The accumulative *csm*, *mat*, and *semi* signals are calculated by applying Eqs 1, 2, and 3 and by using the weights in Table 1. If the genes list still contains the same processed antigen, the DC continuously calculates the accumulative values of the output signals until the *csm* exceeds the threshold.

Finally, the context of the RREQ is determined based on the values of *mat* and *semi*. If *mat* is greater than *semi*, then the RREQ is considered to have originated from an attacker. In this case, the response controller should stimulate the AODV to silently discard the packet. If the *semi* is greater than *mat*, then the RREQ is considered to have been sent by a legitimate node. In this case, the AODV is permitted to continue processing the received RREQ.

## Simulation Discussion

Securing any system entails additional overhead for computational functions. High overhead costs lead to high performance degradation. In this study, a set of experiments are conducted to verify the capability of the MDCA to detect RCAs in MANETs. This section discusses the design of the simulation experiments including the design of network parameters. The efficiency of the MDCA is proved in the results and discussion subsection.

### Simulation Setup

The experiments for testing the MDCA are conducted using Qualnet v5.0.2 [25]. Table 2 lists the fixed values of the parameters employed in all the experiments. The experiments are performed to test the network performance metrics for a MANET employing AODV, MDCA and DCA without any security algorithm. In addition, other set of experiments are performed to test the security performance metrics for AODV performance with MDCA in which iDC size is 1 and DCA, in which the iDC size is 5. Security metrics include FP, FN, and accuracy rates. In the case of a FP, a legitimate node is mistakenly detected as an attacker. In the case of a FN, an attacker is mistakenly detected as a legitimate node. These metrics are complemented by true negative (TN) and true positive (TP) metrics. The calculation for the metrics; FN, FP, TN, TP and accuracy rate; is given in the following Equations:

$$FP=\frac{FP}{TN+FP}$$ (4)

$$FN=\frac{FN}{TP+FN}$$ (5)

$$TN=\frac{TP}{TP+FN}$$ (6)

$$TN=\frac{TN}{FP+TN}$$ (7)

$$accuracy=\frac{TN+TP}{TN+TP+FP+FN}$$ (8)

**Table 2:** Simulation parameters.

Parameter	Value
Network area	1,500 m × 1,500 m
Node speed	0 m/s to 8 m/s
Bandwidth	11 mbps
Packet size	512 bytes (excluding header size)
MAC protocol	802.11
Mobility model	Random way point
Antenna model	Omnidirectional
Path loss model	Two ray
Radio range	250 m
No. of legitimate nodes	30
Simulation time	60 s
No. of attackers	4

The following network performance metrics are also tested: throughput, end-to-end delay, number of retried RREQs, and number of initiated RREPs. Throughput is the number of bits received per unit of time. End-to-end delay is the duration between the time at which the first bit of a packet is sent from the source node side and the time at which the last bit of the same packet is received by the destination side. Performance metrics are measured by varying the radio ranges of RCA attackers (200, 250, 300, 350, and 400 m). All attackers are distributed randomly. Each attacker floods 10 RREQs/s in all simulation runs.

### Results and Discussion

**Fig 7:**
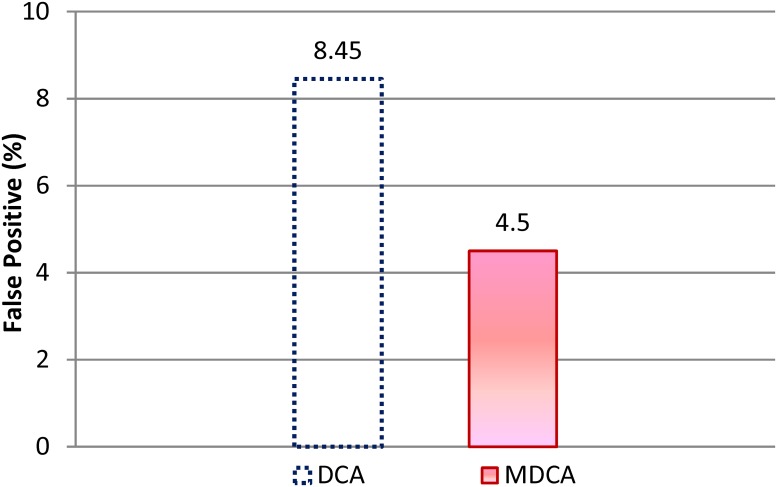
Average FP rates of the MDCA and DCA under RCA.

Fig 7 compares the Average FP rates of the MDCA and DCA under the effect of different attack radio range (200m, 250m, 300m, 350m, 400m). As noticed from the experiments, the FP rate for MDCA and DCA decreases as the attackers’ radio ranges increase. This result is attributed to the attackers’ high coverage and control of the mobile nodes in the MANET when they flood RREQs over wide radio ranges. Therefore, mobile nodes located near or far from the attacker location receives high flooding rate of fake RREQs. Consequently, the percentage of being deceived by fake RREQs decreases. When attackers are within the 200 m radio range, they cannot cover all network nodes. As a result, the nodes in a farther location may receive fake RREQs at a rate lower than 3 and then wrongly consider the attacker as a legitimate node.

Fig 7 also shows that the MDCA outperforms the DCA given the former’s lower FP rates under different attacker radio ranges. The average FP of MDCA is 46.74% lower than the average FP rate recorded by DCA.

**Fig 8:**
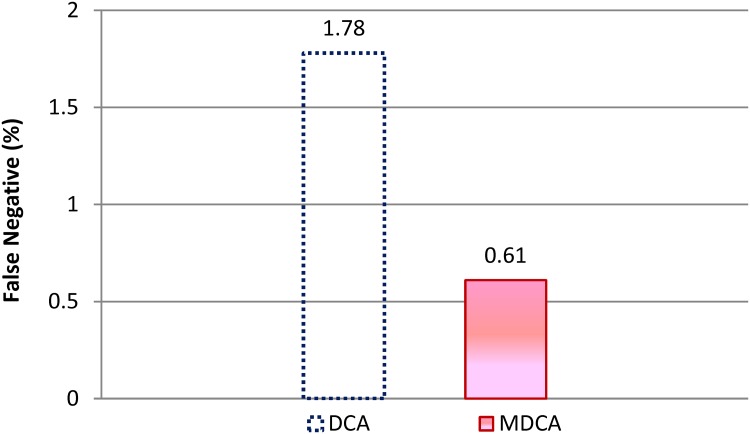
Average FN rates of the MDCA and DCA under RCA.

Fig 8 shows the average FN rate for both MDCA and DCA under the effect of different attack radio range. The attributes of FN rates for both MDCA and DCA are the same as FP rates under the increase of attack radio range. The average FN rate for DCA is higher than that for MDCA by about 65.7%. The high FN rate recorded by the DCA is attributed to the way the algorithm processes input antigens and signals. When iDC processes more than one antigen, the iDC depends on the context of the majority of these antigens to be given to all the processed antigens. This condition leads to high FP and FN rates.

**Fig 9:**
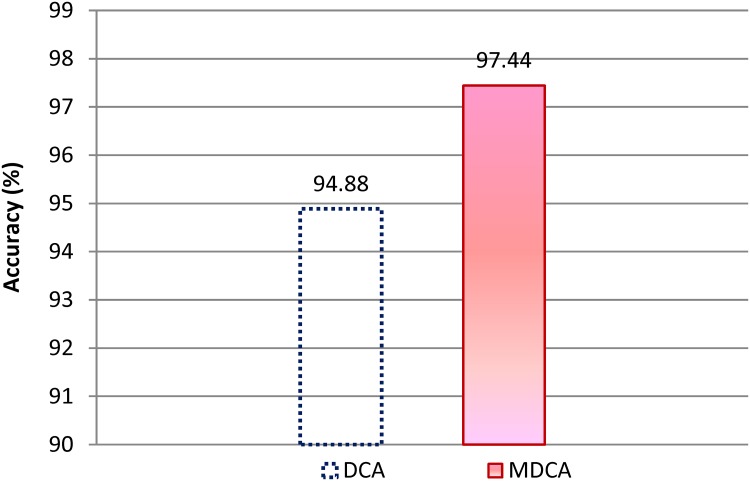
Average accuracy rates of the MDCA and DCA under RCA.

Fig 9 shows the accuracy rates for the MDCA and DCA under different attackers’ radio ranges. As the accuracy of the MDCA and DCA is calculated using Eq 8, their accuracy rates depend on the values of their true positive and true negative rates. Although the accuracy of the DCA appears to be close to that of the MDCA, the MDCA outperforms the DCA under all attackers’ radio ranges.

**Fig 10:**
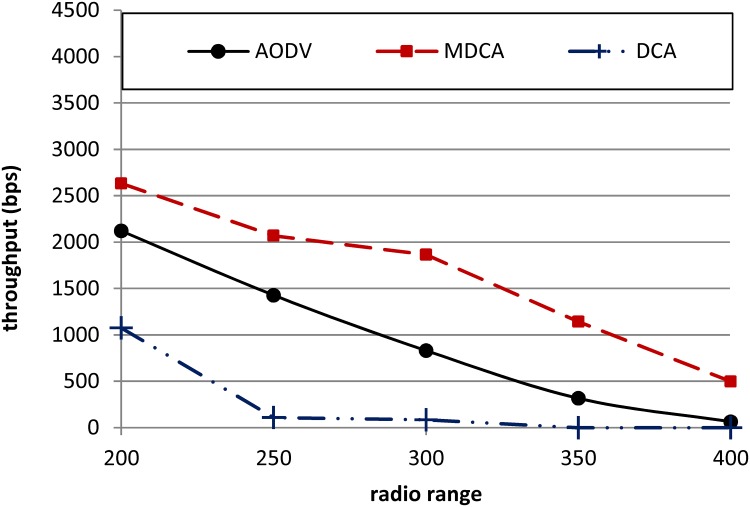
Throughputs of the MDCA, DCA, and AODV under RCA.

Fig 10 shows a comparison of the MDCA, DCA, and AODV under different attackers’ radio ranges. The throughput of the MDCA strongly outperforms that of the DCA and AODV. The throughput of the DCA and AODV are dramatically affected as the attackers’ radio ranges increase. Note that the throughput of the DCA being worse than that of the AODV is attributable to the former’s high FP and FN rates. Although all three curves of the MDCA, DCA, and AODV decrease as the attackers’ radio ranges increase, the MDCA registers the lowest degradation. For example, under the effect of attackers with radio ranges of 400 m (worst case), the throughput of the MDCA is higher than that of the AODV and DCA by 87.2% and 100%, respectively, because the throughput of the DCA degrades to zero under attackers’ radio ranges of 350 and 400 m. The average throughput of the MDCA for all attacker radio ranges is higher than that of the AODV and DCA by about 42% and 100%, respectively. This result proves the resistance of the MDCA under different cases of attacks.

**Fig 11:**
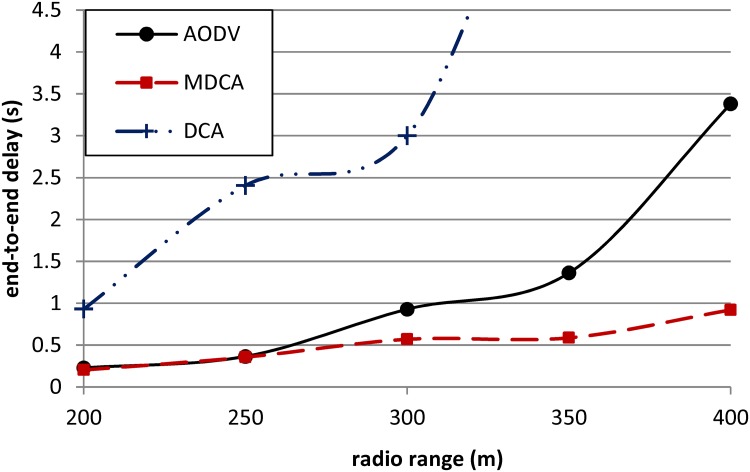
End-to-end delays of the DCA, DCA, and AODV under RCA.

In Fig 11, the end-to-end delay of the MDCA and AODV are similar under attackers’ radio ranges of 200 and 250 m. However, under the effect of attackers’ radio ranges of 300, 350, and 400 m, the end-to-end delay of the AODV clearly increases, whereas that of the MDCA attempts to resist the increment by increasing slowly. Specifically, the end-to-end delay of the AODV under the effect of 400m radio range is higher than that of the MDCA by about 58%. Meanwhile, the DCA exhibits the highest end-to-end delay under the effect of all values of attackers’ radio ranges because of its high FP rate that prevents the initiation of the shortest routes. The end-to-end delay of the DCA does not register under attackers’ radio ranges of 350 and 400 m because the throughputs of the DCA at these points are zero, which indicates that the network is shut down.

The routing overhead represented by initiating the retried RREQs is attributed to the loss of RREQ packets and/or RREP packets when a legitimate source node is trying to discover a route with a legitimate destination node. In normal cases, network congestion can cause packet loss. However, the congestion may be initiated for a certain period and in a certain network location. By contrast, the RCA initiates flooding all over the targeted network thereby extend the duration of the congestion.

**Fig 12:**
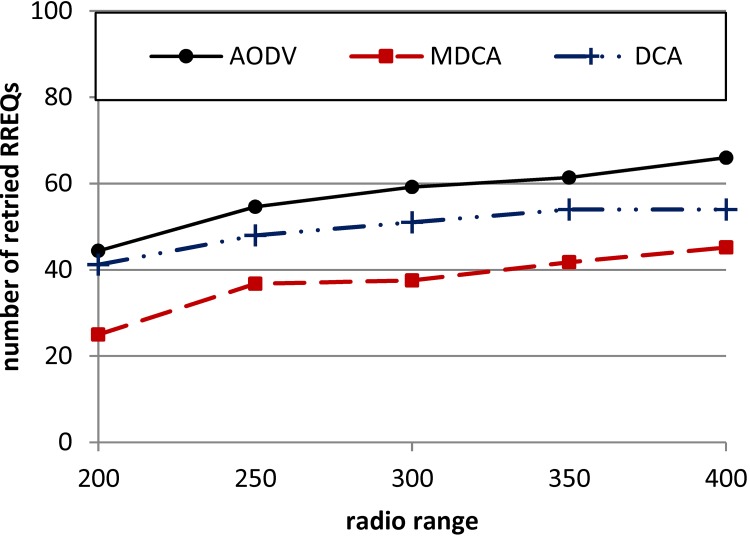
Number of retried RREQs for the MDCA, DCA, and AODV under RCA.

As shown in Fig 12, the AODV shows the highest number of retried RREQs because of the absence of a security algorithm that can disrupt the flooding initiated by the RCA. The second lower number of the retried RREQs is recorded by the DCA, and the lowest values are recorded by the MDCA. The MDCA outperforms the DCA because of its low FP and FN rates. The average number of retried RREQs for the MDCA (over all attackers’ radio ranges) is lower than that for the AODV and DCA by 35% and 25%, respectively.

**Fig 13:**
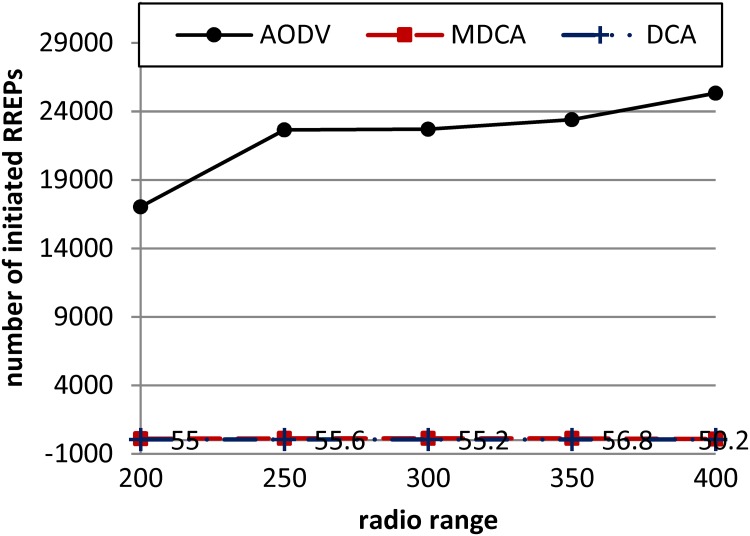
Number of initiated RREPs generated by the MDCA, DCA and AODV under RCA.

Fig 13 compares the MDCA, DCA, and AODV in terms of the routing overhead represented in the number of initiated RREPs generated by each algorithm. The figure shows that the legitimate nodes in the AODV more strongly respond to the RCA compared with the legitimate nodes under the control of the MDCA and DCA. This result proves the capability of both the MDCA and the DCA to suppress the flooding overhead caused by the RCA.

## Conclusion and Future Works

The objective of this study is to investigate the capability of a danger theory-based artificial immune algorithm, that is, the MDCA, to detect flooding-based attacks, representing by RCA, in MANETs. The MDCA applies the DCA to secure MANETs with additional improvements. In this study, the MDCA was tested and validated using Qualnet v7.1 simulation tool. A new simulation module of a flooding attack referred to as the RCA was introduced using Qualnet v7.1. The MDCA outperformed the DCA in terms of security and network measurements. The MDCA also demonstrated high robustness and resistance to RCA flooding. In addition, the MDCA successfully secured the MANET without affecting its network performance. On the basis of the comparison between the MDCA (iDC size = 1) and DCA (iDC size = 5), we can conclude that a high number of antigens processed in iDC leads to a low performance for the security algorithm.

In the future, we will test the performance of the MDCA given different numbers of attackers with different attack positions in the MANET. Real test bed experiments will be conducted to test the feasibility of applying the MDCA in MANETs.
